# Supplementary material for: Structural basis of Retron–Eco8-mediated antiphage defense
Source: Nucleic Acids Res. 2026 Feb 10;54(4):gkag111. doi: 10.1093/nar/gkag111 (PMC12887533; doi:10.1093/nar/gkag111)
Supplement: gkag111_Supplemental_File [file gkag111_supplemental_file.pdf]

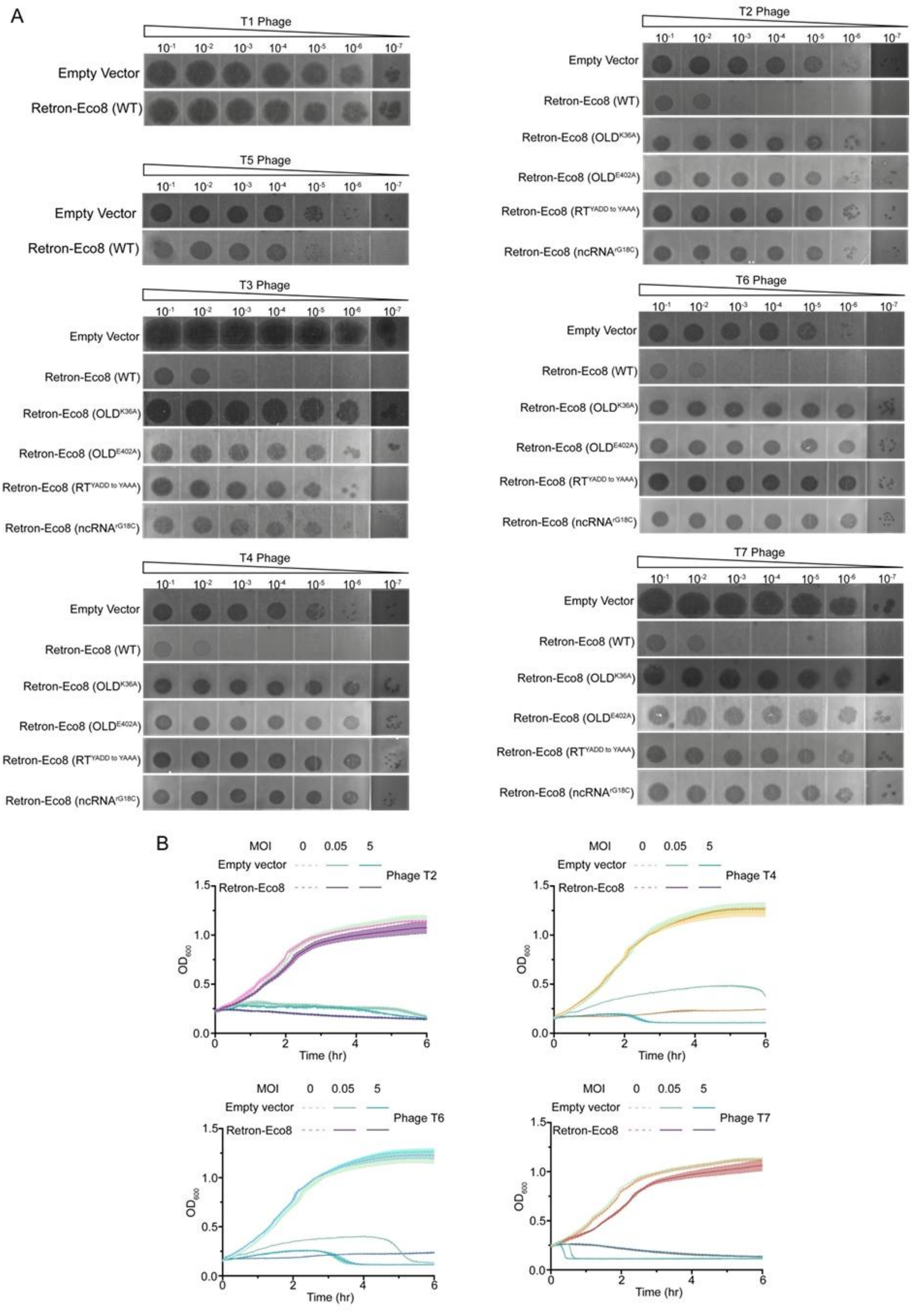

Supplementary Figure 1. The anti-phage activities of Retron-Eco8 system against various phages. Empty vector is used as the negative control. (A) Plating assays of various phages on Retron-Eco8 system. **(B)** Phage infection in liquid cultures of the *E. coli* BL21(DE3) strain containing Retron-Eco8. Cells are infected at various MOI values. For each MOI, results of three experiments are presented as the average of three replicates with shaded areas indicating SD.

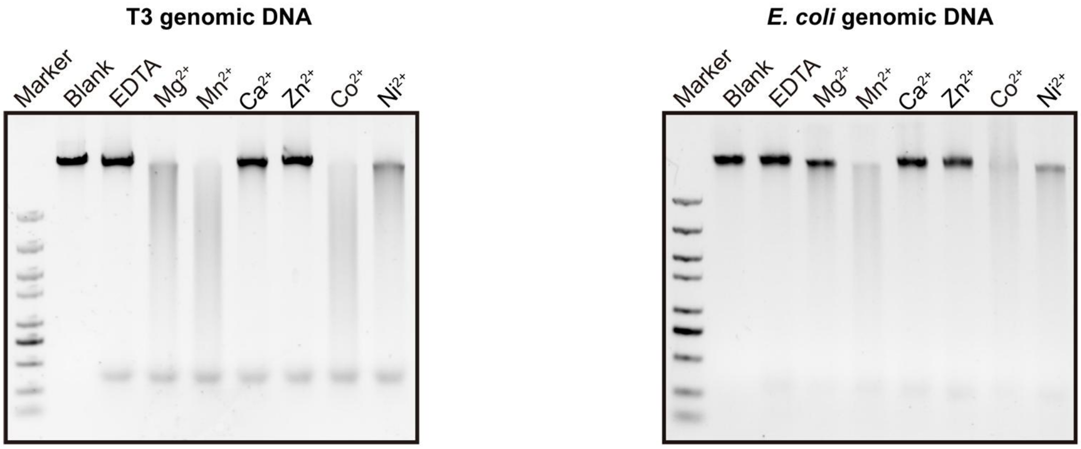

Supplementary Figure 2. The metal dependent nuclease activity of Retron-Eco8 against T3 phage and *E. coli* genomic DNA.

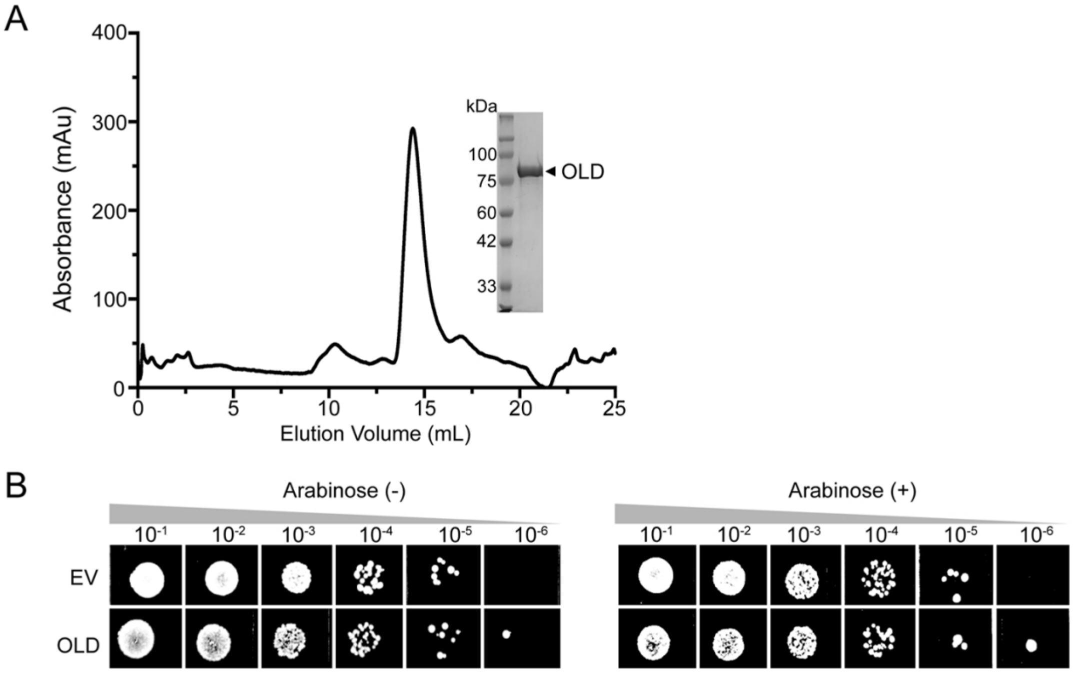

Supplementary Figure 3. Over-expression of OLD alone does not demonstrate cytotoxicity. (A) Gel-filtration profile for OLD alone. The elution peak is evaluated by SDS-PAGE. **(B)** Plating assay for the cytotoxicity of OLD alone. The empty vector (EV) is used as the negative control.

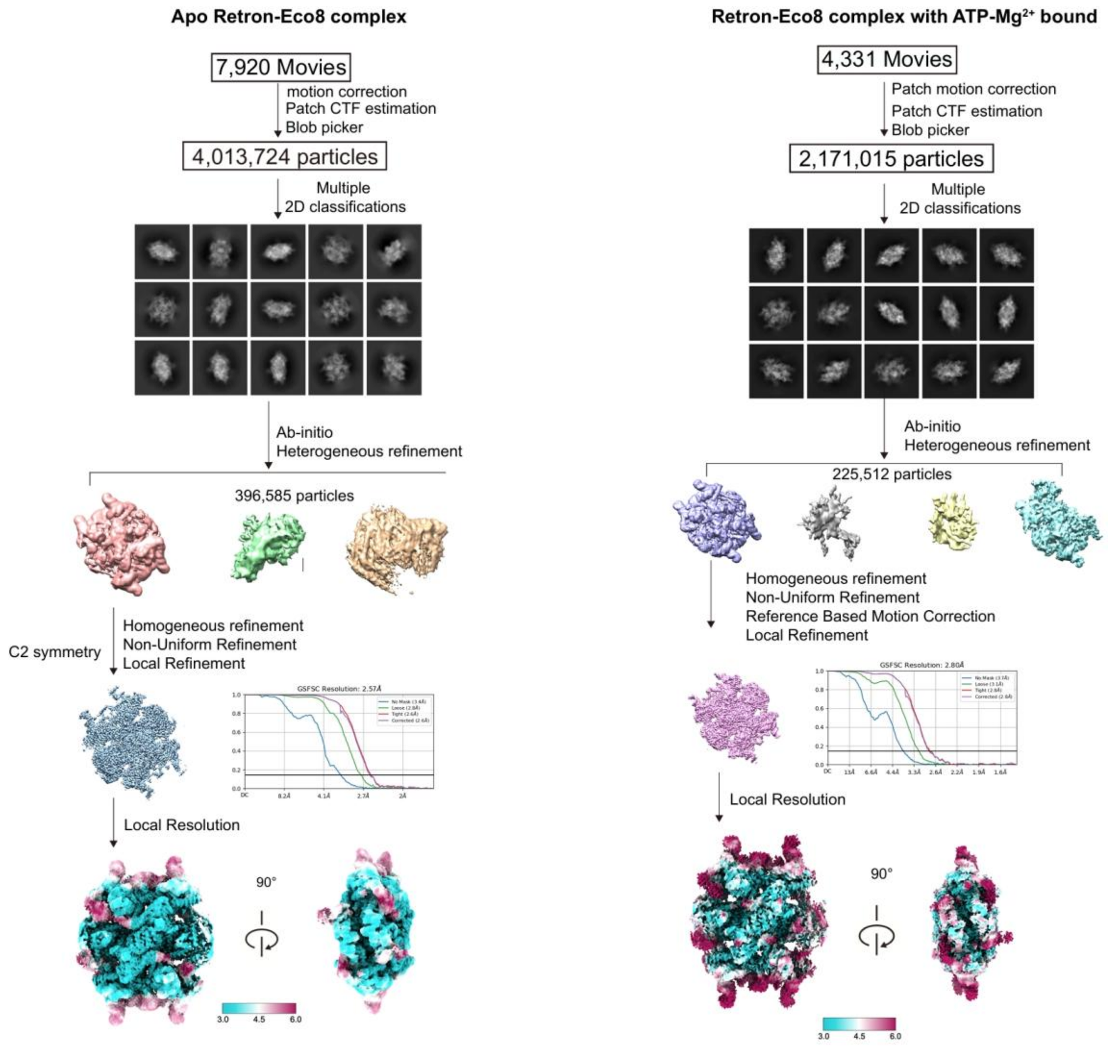

Supplementary Figure 4. Single particle cryo-EM analysis of Retron-Eco8 and Retron-Eco8 complexed with ATP–Mg^{2+}. Representative cryo-EM micrographs, representative reference-free 2D-class averages, and data-processing workflows for Retron-Eco8 in the indicated states.

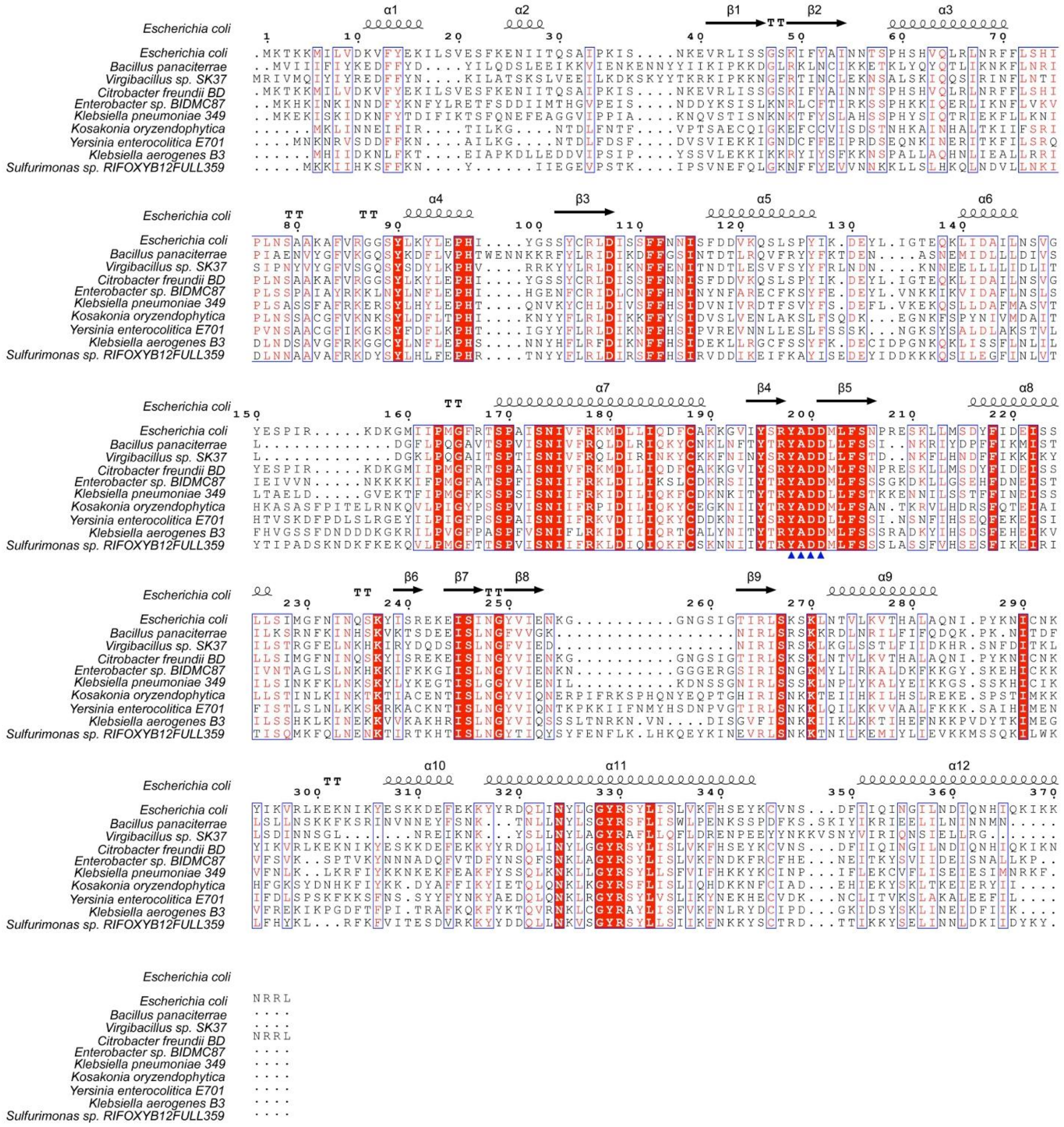

Supplementary Figure 5. Sequence alignment of Retron-Eco8 RTs of various species. The secondary structure elements of *E. coli* Retron-Eco8 RT are shown above the alignment. The catalytic YADD motif is indicated by blue triangles.

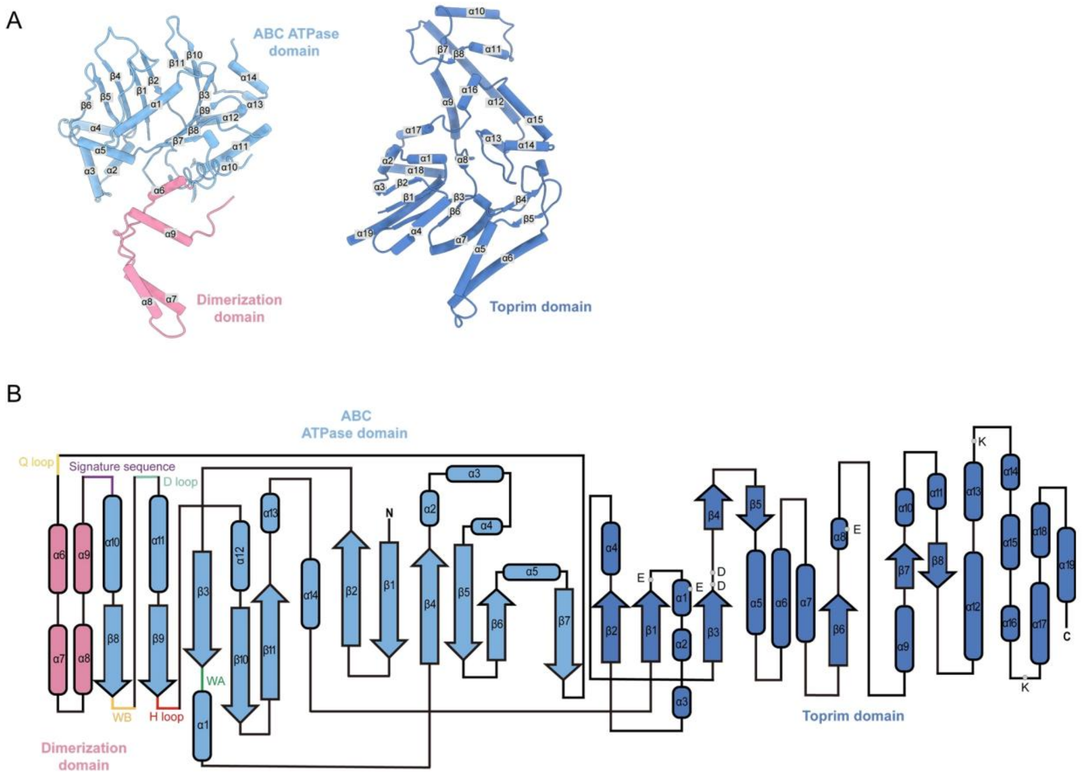

Supplementary Figure 6. Architecture of Retron-Eco8 OLD. (A) The secondary structures of the ATPase, Dimerization and Toprim domains of Retron-Eco8 OLD. **(B)** Topology diagrams of Retron-Eco8 OLD. Six conserved motifs in the ATPase domain and the key residues in the Toprim nuclease active are labelled.

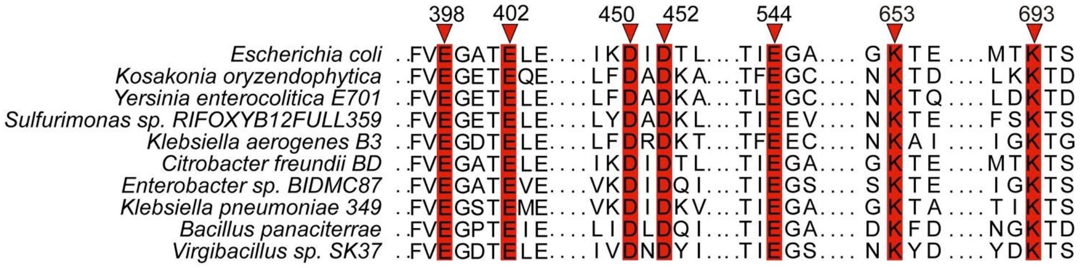

Supplementary Figure 7. Sequence alignment reveals that the key residues in Toprim domain active site are conserved among various species.

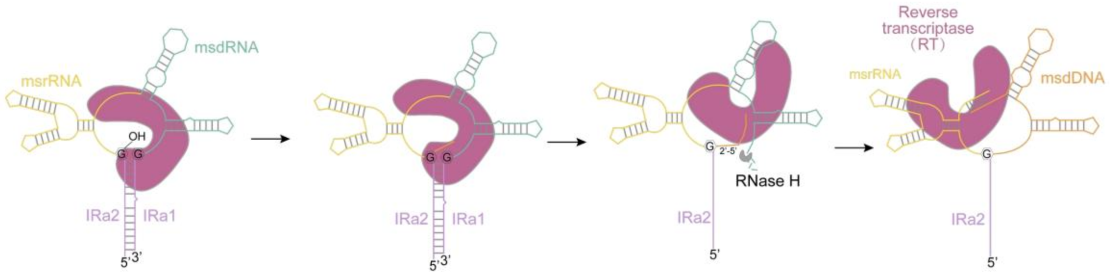

Supplementary Figure 8. Schematic depiction of the retron msDNA synthesis process using Retron-Eco8 as an example.

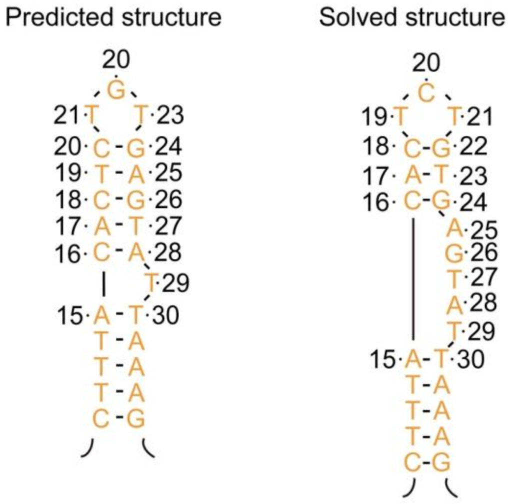

Supplementary Figure 9. Comparison of the predicted and solved DSLa structures of Retron-Eco8 msDNA.

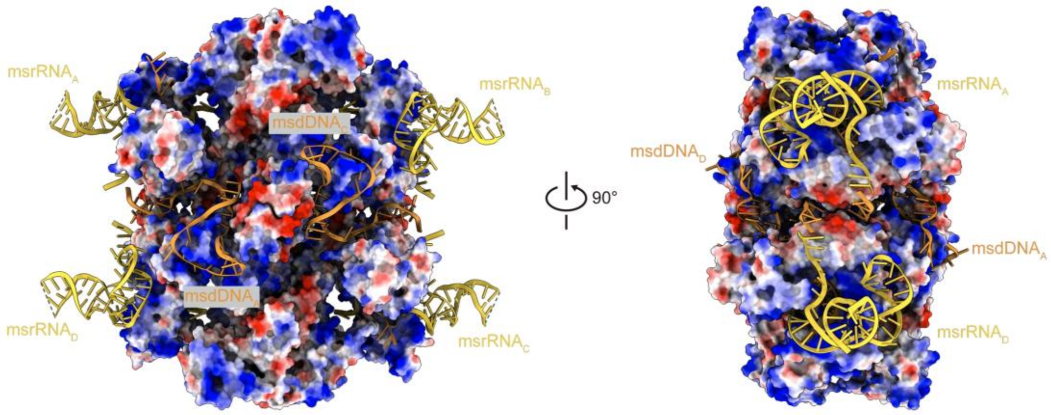

Supplementary Figure 10. Retron-Eco8 msDNA wraps the positively charged surfaces of RT and OLD. The electrostatic surface potential of Retron-Eco8 RT and OLD is shown. Blue and red (+/-5 kT/e) indicate the positively and negatively charged areas, respectively.

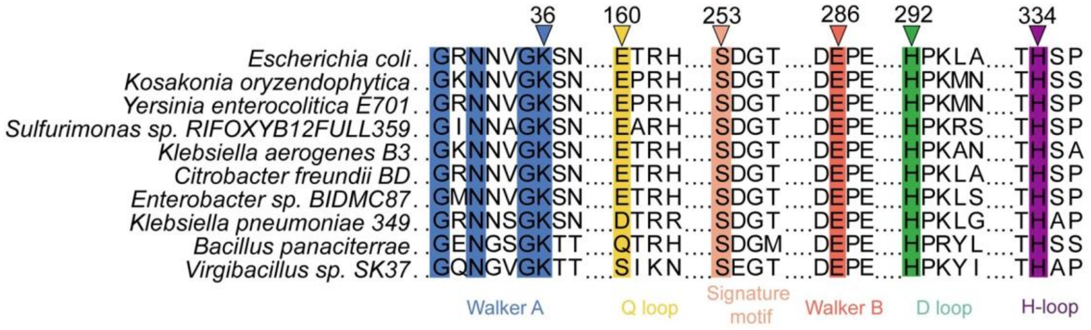

Supplementary Figure 11. Sequence alignment of Retron-Eco8 OLD ATPase domain of various species demonstrates the conserved key residues in ATPase active site.

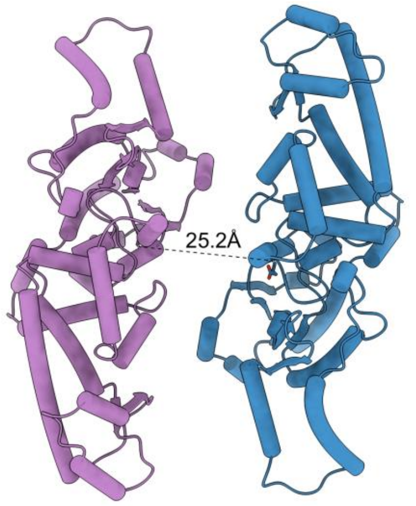

Supplementary Figure 12. the distance between the Cα atoms of catalytic residue E402 in adjacent OLD protomers. The E402 residues are shown in stick.

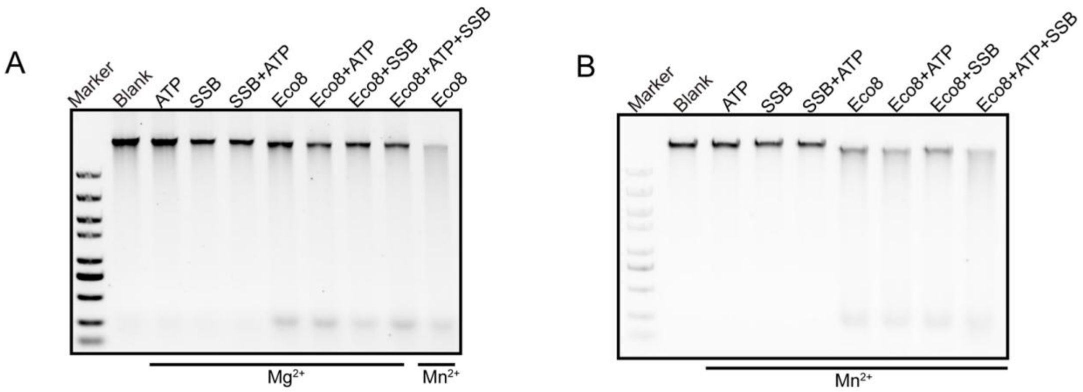

Supplementary Figure 13. Effects of divalent cations or T7 SSB on Retron-Eco8 nuclease activity towards E. coli genomic DNA. (A) Retron-Eco8 exhibits negligible *in vitro* nuclease activity under Mg^{2+} conditions. Mn^{2+} is used as the positive control. **(B)** The presence of T7 SSB does not significantly enhance the *in vitro* nuclease activity of Retron-Eco8.

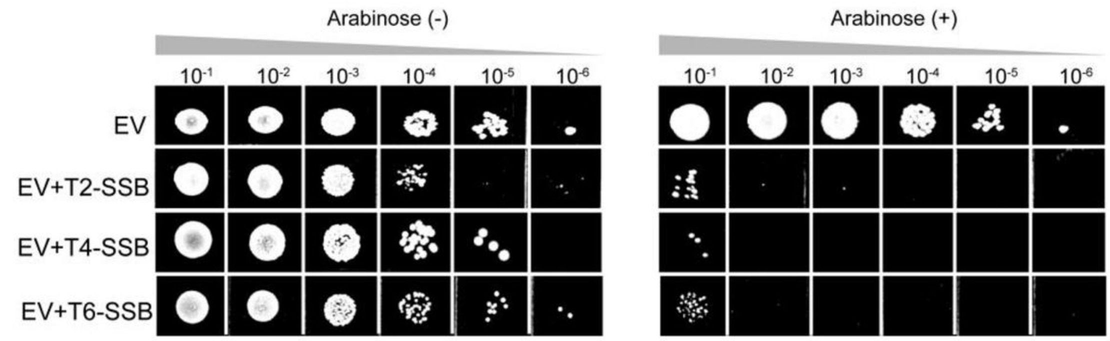

Supplementary Figure 14. Representative plating assay shows that over-expression of phage SSB proteins induces cytotoxicity. Empty vector (EV) is used as the negative control.

Supplementary Table S1. Cryo-EM data collection, refinement, and validation statistics

Structure	Retron-Eco8	Retron-Eco8 (ATP-Mg^{2+})
EMDB ID	66659	66663
PDB ID	9X94	9X9B
**Data collection and procession**		
Magnification	165000	165000
Voltage (kV)	300	300
Electron exposure ($e^-/Å^2$)	56.58	50
Defocus range (μm)	-1.1~-1.8	-0.8~-1.4
Pixel size (Å)	0.85	0.75
Symmetry imposed	C2	C1
Initial particle images (no.)	4,013,724	2,171,015
Final particle images (no.)	3,030,229	123,608
Map resolution (Å)	2.57	2.8
FSC threshold	0.143	0.143
Map resolution range (Å)	2.213-24.25	2.418-8.141
Map sharpening *B* factor ($Å^2$)	-74.2	-63.6
**Refinement**		
Initial model used (Protein)	Alphafold3	Alphafold3
Initial model used (Nucleotides)	de novo	de novo
Model resolution (Å)	2.8	3.0
FSC threshold	0.5	0.5
**Model composition**		
Non-hydrogen atom	44,906	45,453
Protein residues	4,396	4,445
Nucleotide	422	424
Ligand	-	188
**B-factor ($Å^2$)**		
Protein	42.65	71.43
Nucleotide	60.37	90.50
Ligand		56.39
**R.m.s. deviations**		
Bond lengths (Å)	0.008	0.006
Bond angles (°)	0.827	0.736
**Validation**		
MolProbity score	1.70	1.87
Clashscore	6.55	6.39
Poor rotamers (%)	0.46	0.12
**Ramachandran plot**		
Favored (%)	95.15	90.81
Allowed (%)	4.83	9.19
Outliers (%)	0.02	0.00
